# Evaluation of retinal ganglion cell function after intraocular pressure reduction measured by pattern electroretinogram in patients with primary open-angle glaucoma

**DOI:** 10.1007/s10633-017-9575-0

**Published:** 2017-02-07

**Authors:** Joanna Karaśkiewicz, Krzysztof Penkala, Maciej Mularczyk, Wojciech Lubiński

**Affiliations:** 1 Department of Ophthalmology, Pomeranian Medical University, Powstańców Wielkopolskich 72 Str., Szczecin, Poland; 2 Department of Systems, Signals and Electronics Engineering, Faculty of Electrical Engineering, West Pomeranian University of Technology, Szczecin, Poland; 3 Department of General and Clinical Anatomy, Pomeranian Medical University, Szczecin, Poland

**Keywords:** POAG treatment, Retinal ganglion cell function, Pattern electroretinogram

## Abstract

**Background:**

To evaluate retinal ganglion cell (RGC) function after intraocular pressure (IOP) reduction measured by pattern electroretinogram (PERG) in patients with newly diagnosed, non-treated preperimetric and early stages of primary open-angle glaucoma (POAG).

**Methods:**

Twenty-four eyes from 24 patients with POAG: 11 eyes with preperimetric glaucoma and 13 eyes with early glaucoma received *Ganfort* ^®^ (bimatoprost + timolol) once a day for a period of 1 month. Before and after the treatment, following measurements were analyzed: IOP, mean ocular perfusion pressure (MOPP), peak time of P50 and amplitude of P50 and N95 waves in PERG (ISCEV standard 2012). Correlations between PERG P50 and N95 waves, IOP and MOPP were calculated.

**Results:**

After therapy, IOP significantly decreased in all eyes, on average 31%. Significant increase in MOPP in all eyes on average 14% was detected. PERG amplitude of P50 and N95 waves increased in 75 and 79% eyes, respectively, on average P50 by 28% and N95 by 38%. There were no significant interactions between the change of PERG parameters in time and stage of glaucoma.

**Conclusions:**

Significant IOP-lowering therapy can improve RGC function measured by PERG, in patients with preperimetric and early stages of POAG.

## Introduction

Glaucoma is described as an optic neuropathy, in which retinal ganglion cells (RGCs) degenerate and, in consequence, a progressive loss of vision can occur [1]. For many years, glaucomatous damage of the optic nerve was considered irreversible, but as many research results show, this theory is false: appropriate therapy lowering intraocular pressure (IOP) can improve not only changes in the visual field [2], but also the function of RGC, which can be measured by electrophysiological tests, such as pattern electroretinogram (PERG) [3] or PERG optimized for glaucoma screening (PERGLA) [4]. It is well known that the optimal IOP reduction depends on the degree of visual field damage at diagnosis and the rate of progression [1]. There is no single and safe IOP level. It varies between patients and eyes. That is why the ‘target IOP’ is the highest IOP expected to prevent further glaucomatous damage or slow down disease progression to a minimum [1].

Dietlein et al. [5] tried to evaluate the percentage of IOP that should be reduced from its baseline value. They recommend a reduction by 20–50%, depending on the degree of existing damage, the baseline IOP, the rate of progression and, finally, patient’s age. According to their review, topical monotherapy can decrease IOP between 15 and 30%, while IOP reduction observed after incisional glaucoma surgery ranges from 50 to 90%. However, we still do not know how much IOP should be decreased in order to improve RGC function.

A clinical trial published in 2011 by Sehi et al. [6] tried to evaluate the impact of IOP reduction on RGC function in glaucoma suspect and glaucomatous eyes receiving latanoprost 0.005%, using PERGLA. This prospective trial did not reveal significant changes in PERGLA amplitude after therapy, with either latanoprost or placebo. According to authors, one of the most important reasons of such results is that mean reduction in IOP after latanoprost (20 ± 13%) was insufficient to improve the signal generated by RGC.

The results of another study performed by Sehi et al. [7] made likely that after surgical reduction in IOP (which was about 47%), reversal of RGC dysfunction occurs and may be quantified using PERGLA.

That is why we presumed that, to improve the RGC function, IOP should be decreased 30% or more.

Although there are no clear guidelines which group of medications should be the first choice treatment, prostaglandin analogs are often used as the first choice drugs, because they are effective, comfortable in using (once daily) and have a minimal risk of hyperemia. However, many patients require more than one medication to achieve target IOP. Nowadays, the most commonly used drug added to prostaglandins is a beta-blocker, such as timolol [8].

According to a meta-analysis [8], evaluating the IOP-lowering effects and tolerability of the three prostaglandin–timolol fixed combinations (PG–timolol FCs), IOP reduction was significantly greater with bimatoprost–timolol FC, compared to latanoprost–timolol FC and travoprost–timolol FC. The incidence of hyperemia was not significantly lower with latanoprost–timolol FC than with bimatoprost–timolol FC.

It seemed sensible to hypothesize that Ganfort^®^ (Allergan Pharmaceuticals Ireland), which contains bimatoprost (0.3 mg/ml) and timolol maleate (5 mg/ml), could be the medication that will improve RGC function, because it reduces IOP by 35.1% [9].

The clinical significance of a 30% IOP reduction is based on observation from major trials [10–14] as well as on new glaucoma guidelines [15]. This reduction can stop the progression of glaucoma neuropathy. The aim of this study was to evaluate the influence of recommended IOP reduction on RGC function, measured by PERG in eyes of patients with non-treated preperimetric or early stages of primary open-angle glaucoma (POAG). We could expect that, in these stages of disease, RGCs are not only apoptotic and/or with structural changes, but some are just dysfunctional [16]. By lowering IOP, we may restore RGC function and prevent apoptosis [17]. The transient PERG was used to check whether this commonly used stimulation is enough sensitive to monitor changes of RGC function after IOP-lowering therapy.

## Methods

### Subjects

Twenty-four eyes from 24 patients aged 56 ± 11 years with newly diagnosed preperimetric or early stages of POAG, without previous treatment, were enrolled in the study. They were recruited from ophthalmological outpatient departments. The preperimetric POAG was diagnosed, according to actual criteria: a glaucomatous appearance of the optic disc head, open chamber angle, and thinning of retinal nerve fiber layer (RNFL) thickness, measured by scanning laser polarimetry (GDx) or optical coherence tomography (OCT), with normal visual field (VF), confirmed by at least two following standard automated perimetry (SAP) (HFA 24-2 W-W/SITA standard strategy) examinations [18]. The early stage of POAG was diagnosed, according to the European Glaucoma Society, on the basis of SAP, and each SAP was repeated once to confirm VF abnormalities. All patients underwent a complete ophthalmic examination: distance best-corrected visual acuity (DBCVA) using logMAR-ETDRS chart, slit lamp biomicroscopy, gonioscopy, Pascal applanation tonometry, dilated stereoscopic fundus examination (VOLK 90) and PERG. Blood pressure (BP) of all patients was measured with the use of a blood pressure device to calculate mean ocular perfusion pressure (MOPP), defined as the difference between 2/3 mean arterial pressure (MAP) and IOP.

All abovementioned examinations were performed twice: before treatment and 1 month after *Ganfort* instillation every day at 7 p.m. Patients’ characteristics are shown in Table 1.

**Table 1:** Groups characteristic: preperimetric and early-stage POAG

Stage of glaucoma	Trait	*n*	Mean	SD	Min	Max
Early	Age (y)	13	61.2	8.06	39.0	72.0
	DBCVA (logMAR)	13	0.05	0.06	0.15	0.00
	IOP1 (mmHg)	13	24.57	6.48	15.90	37.20
	MOPP1	13	42.99	9.81	25.38	57.87
Preperimetric	Age (y)	11	50.64	12.44	22.00	66.00
	DBCVA (logMAR)	11	0.06	0.06	0.15	0.00
	IOP1 (mmHg)	11	23.90	4.98	16.90	34.90
	MOPP1	11	41.66	6.20	28.98	47.63

*DBCVA* distance best-corrected visual acuity, *IOP1* intraocular pressure before treatment, *MOPP1* mean ocular perfusion pressure before treatment, *y* years, *n* number of eyes, *SD* standard deviation

Exclusion criteria were as follows: DBCVA <0.15, corneal/retinal pathology, age <35 or >75, prior intraocular surgery (except for uncomplicated cataract surgery), unreliable SAP (>33% rate of fixation loses), contraindications to bimatoprost and/or timolol and/or PERG, ocular or systemic disease with a known influence on retinal function (ex. diabetes mellitus, dementive diseases) at the time of diagnosis or throughout the follow-up.

This project was authorized by the Local Ethical Committee of the Pomeranian Medical University in Szczecin. An informed written consent was obtained from all subjects after the purpose of the study, and possible risks were clarified.

### Electroretinography

Transient PERG was recorded with the RetiPort (Roland Consult GmbH, Germany) system according to the ISCEV standard 2013 [19]. Monocular stimulation was used without pupil dilation; refraction correction was applied with respect to the eye-screen distance. Central fixation was used. The patient was monitored with a TV camera, and interruptions of the test were introduced when fixation loss or frequent blinking was observed. 21″ CRT monitor with a frame rate of 70 fps was used for pattern stimulation; dimension of the stimulus field was 15°24′ (the mean of the width and the height of the screen), with the aspect ratio between the width and the height (screen proportion H/V) equal to 4:3; black and white reversing checkerboard was presented to the patient, with a check size equal to 0°48′; luminance for white elements was equal to 118 cd/m^2^, mean luminance of the stimulus screen: 60 cd/m^2^, with Michelson contrast set to 97%; and temporal frequency was equal to 4.0 rps (2.0 Hz). Thread DTL-like electrode (Roland Consult) was used as active, gold disc (Grass, USA) skin electrode was placed at the ipsilateral outer canthus as reference, and ground (gold disc, Grass) electrode was placed on the forehead (Fpz). Parameters of the recording channel were as follows: amplifiers sensitivity 20 µV/div, filter frequency bandwidth 1–100 Hz. The analysis period (sweep time) was equal to 250 ms. Artifact rejection threshold was set to 95% (for the amplifiers range ±100 µV), and 200 sweeps were averaged. For each eye, two consecutive PERG waveforms were recorded and off-line averaged for further analysis. According to the standard, amplitude of P50 and N95 waves, as well as peak time of P50 wave were analyzed. Values of all parameters were compared with the laboratory own normal values.

The first author had good experience in using DTL electrode for PERG recording.

### Statistical analysis

The descriptive statistics such as the mean value, the standard deviation, the minimum and maximum values of age, DBCVA, IOP and MOPP before treatment in the groups in terms of the stage of glaucoma were calculated. The assumption of normality was verified using the Shapiro–Wilk test. The obtained parameters were analyzed using mixed model ANOVA (factorial repeated measures ANOVA). The following parameters: PERG amplitude, peak time, IOP and MOPP, were treated as dependent variables and the stage of glaucoma as a categorical factor. It was determined to change each parameter at two time points before and after therapy and the interaction of changes over time depending on the groups (stage of glaucoma). Correlations between selected parameters were investigated using Pearson coefficient. Results were considered significant with *p* < 0.05. Data were analyzed using STATISTICA 12.5 software.

## Results

**Fig. 1:**
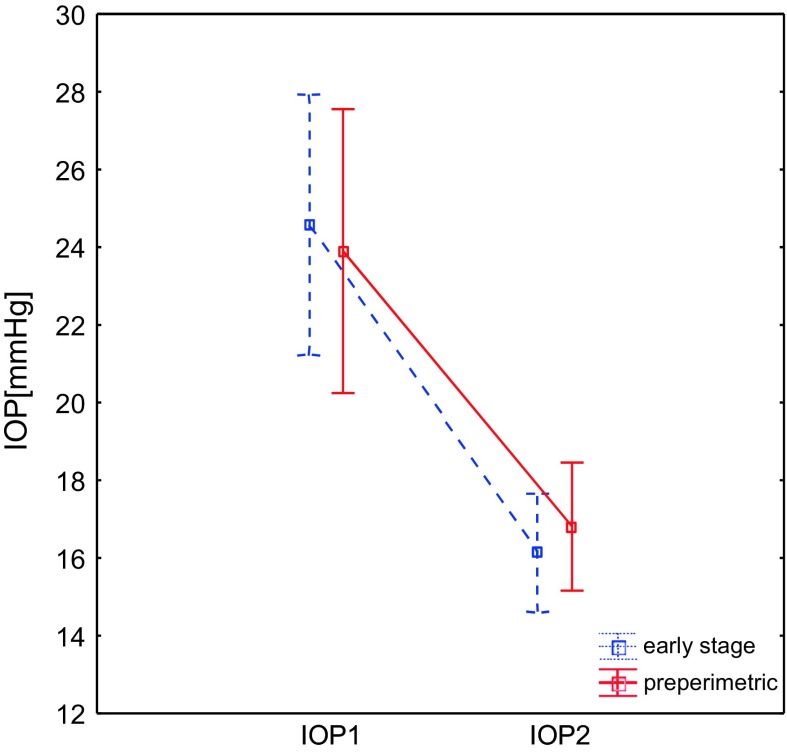
Changes of the mean values and its standard errors (*whiskers*) of the IOP before (*1*) and after (*2*) therapy. *IOP* intraocular pressure

**Fig. 2:**
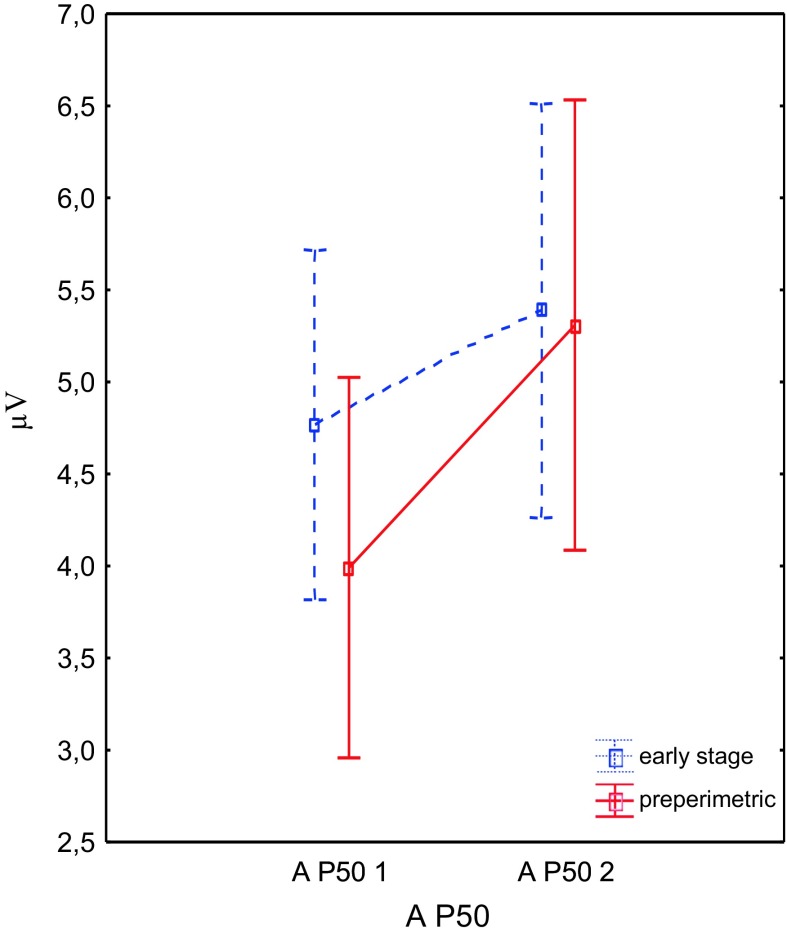
Changes of the mean values and its standard errors (*whiskers*) of the P50 amplitude before (*1*) and after (*2*) therapy. *A* amplitude

**Fig. 3:**
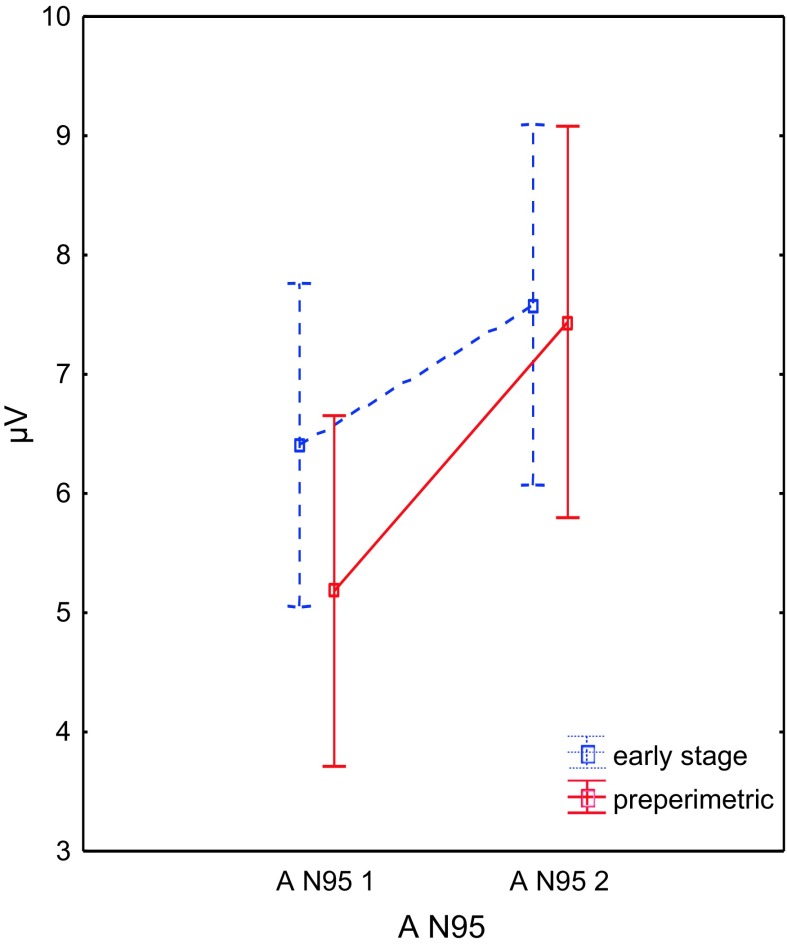
Changes of the mean values and its standard errors (*whiskers*) of the N95 amplitude before (*1*) and after (*2*) therapy. *A* amplitude

The applied therapy significantly decreased IOP in all eyes, on average 31%. In Fig. 1 changes of the mean values and its standard errors (whiskers) of the IOP before and after therapy are shown. The change of the IOP was significant for both groups, although there was not significant effect between groups. Mean ocular perfusion pressure increased in all eyes on average 14%. Figures 2 and 3 present changes of the mean values and standard errors of P50 and N95 amplitudes. In Table 2, the mean values before and after treatment of IOP, MOPP, and PERG amplitudes of P50 and N95 waves and peak time (PT) of P50 wave are shown. Significant changes were observed in all parameters, with an exception of PT P50.

**Table 2:** Values of the examined parameters before and after therapy and the *p* value of the factorial repeated measures ANOVA

Parameter	Mean ± SD	*n*	Difference ± SD	Mean % of change	*p*
A P50 1 (µV)	4.4 ± 1.66	24	−0.94 ± 1.54	27.77	0.0055
A P50 2 (µV)	5.4 ± 1.91				
A N95 1 (µV)	5.8 ± 2.38	24	−1.67 ± 1.90	38.28	0.0002
A N95 2 (µV)	7.5 ± 2.57				
PT P50 1 (ms)	51.8 ± 3.29	24	−0.43 ± 1.99	1.00	0.3428
PT P50 2 (ms)	52.2 ± 2.48				
IOP 1 (mmHg)	24.3 ± 5.72	24	7.82 ± 4.52	30.51	0.0000
IOP 2 (mmHg)	16.4 ± 2.60				
MOPP 1	42.4 ± 8.21	24	−5.02 ± 7.18	14.25	0.0031
MOPP 2	47.4 ± 7.22				

*A* amplitude, *PT* peak time, *IOP* intraocular pressure, *MOPP* mean ocular perfusion pressure, *n* number of eyes, *SD* standard deviation

PERG amplitudes of P50 and N95 waves increased in 75 and 79% eyes, respectively, on average P50 by 28% and N95 by 38%.

The improvement in RGC function, defined as 20% or more increase in at least one of the PERG waves, was noticed in 71% of eyes (17/24). In 29% of eyes (7/24), the PERG amplitudes did not improve.

We did not find any statistical significant correlations between the change of the parameters in time and stage of glaucoma (Table 3).

**Table 3:** Results of the factorial repeated measures ANOVA, showing non-interaction between change of the following parameters in time and stage of glaucoma

Interaction	*F*	*p*
A P50	1.226	0.2801
A N95	2.034	0.1679
PT P50	0.897	0.3538
MOPP	0.164	0.6896
IOP	0.512	0.4820

*A* amplitude, *PT* peak time, *IOP* intraocular pressure, *MOPP* mean ocular perfusion pressure

**Fig. 4:**
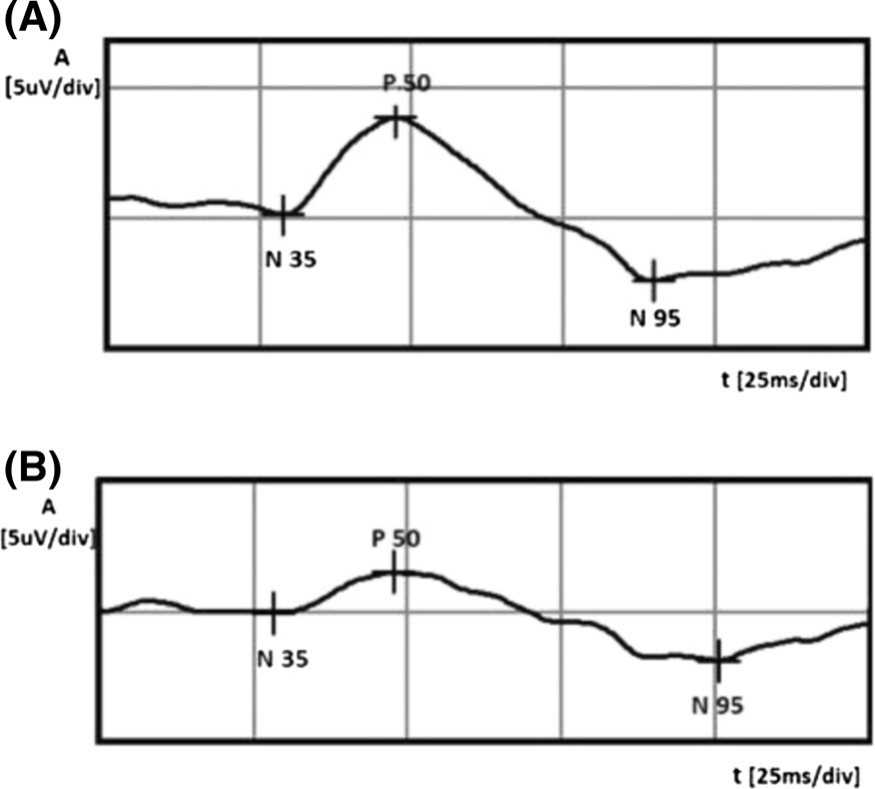
Example of PERG amplitude of P50 and N95 waves improvement after treatment (**A** after treatment, **B** before therapy). *A* amplitude, *t* time

For example, PERG examination before and after treatment from an eye of a 56-year-old woman with an early stage of POAG is shown in Fig. 4. Significant improvement in P50 and N95 waves after therapy is observed.

The characteristics of eyes before and after therapy are presented in Table 4.

**Table 4:** Characteristics of eyes before and after treatment

Eye	Before therapy					After therapy				
	IOP (mmHg)	AP50 (µV)	AN95 (µV)	PT P50 (ms)	MOPP	IOP (mmHg)	AP50 (µV)	AN95 (µV)	PT P50 (ms)	MOPP
1	33.5	3.68	3.55	47.5	33.39	14.6	4.55	6.26	50.9	48.73
2	19.6	4.41	6.39	51.4	51.73	12.9	6.79	8.86	51.9	48.43
3	23	3.36	4.37	53.3	56.78	16.2	4.74	6.82	55.8	66.47
4	19.4	3.82	5.15	54.8	34.82	14.3	5	7.03	52.3	45.03
5	24.8	2.26	3.39	53.8	41.64	18.2	3.38	4.5	55.3	50.24
6	19.6	7.37	10.4	53.3	36.40	13.5	8.11	11.4	52.8	47.17
7	16.9	5.14	7.19	51.4	35.54	12.9	9.35	13.2	50.9	39.77
8	21	2.57	3.34	59.7	46.33	13.3	3.5	4.97	59.2	45.37
9	28.8	4.47	7.51	49.9	28.98	18.7	6.89	9.08	51.4	37.08
10	23.5	7.66	9.02	52.3	43.39	15.2	6.68	9.63	51.4	42.80
11	24.7	5.27	6.62	50.9	44.19	14.6	5.19	7.22	53.3	44.73
12	25.9	2.86	2.29	54.8	39.88	19	4.69	6.52	52.8	42.78
13	21.5	1.32	3.09	49.9	47.39	14.8	2.15	5.5	50.9	52.31
14	34.6	5.06	7.17	52.3	46.07	21.8	7.71	10.3	53.8	50.20
15	24.8	5.71	6.92	56.3	57.87	19.8	8.38	10.7	52.3	42.42
16	15.9	3.44	5.14	50.4	44.10	13	3.13	4.63	50.9	49.00
17	21.9	6.62	10.1	50.4	42.54	17.5	6.43	7.87	51.9	54.28
18	34.9	4.46	5.05	50.9	46.66	20.5	4.52	7	51.4	59.50
19	25.7	4.27	4.32	51.9	47.63	19	3.08	4.32	51.9	53.44
20	22.4	6.4	8.91	48.4	25.38	18	5.79	8.81	47.5	30.67
21	20.7	6.4	7.7	51.9	43.74	15.7	3.89	5.95	51.9	49.19
22	21.3	2.92	3.1	53.3	46.92	16.1	4.83	4.58	51.4	42.34
23	20.7	3.47	6.65	42.1	46.41	17.7	6.48	11	47	47.86
24	37.2	2.94	2.95	52.5	29.24	17.4	3.19	4.21	54.8	47.71

*IOP* intraocular pressure, *MOPP* mean ocular perfusion pressure, *A* amplitude, *PT* peak time

## Discussion

The results of this study suggest that approximately 30% IOP reduction in eyes of patients with preperimetric and early stages of POAG significantly improves function of RGC, as registered by the PERG test.

In evaluating the activity of RGCs before and after treatment, we used PERG, because it can measure reversal of RGC dysfunction in glaucomatous eyes that undergo pharmacological reduction in IOP [3, 20]. This electrophysiological test reflects not only RGC apoptosis, but also the dysfunction of other RGCs that may be reversible, at least in part, after proper IOP-lowering therapy [20]. The definition of improvement was framed on the basis of the data from the literature concerning reproducibility of PERG: according to Otto and Bach [21], the coefficient of variation (CV) for transient PERG is 12 ± 2%. That is why we assumed that, if at least one of the PERG waves increases after treatment over 20% of the baseline value, it would strongly suggest an improvement in RGC function. It is in accordance with other studies [22].

Different mechanisms with an influence on RGC’ function recovery should be considered. To better understand the role of IOP-lowering therapy, glaucomatous neurodegeneration must be considered as a product of multiple factors [23]. Although there are a number of hypotheses, the entire understanding of glaucoma pathogenesis has not yet been gained. It is a combination of 1—axonal insult in the optic nerve head (ONH) [24], 2—diminished retrograde delivery of neurotrophins [25], 3—impaired glial cell interactions [26] and 4—local inflammatory response of astrocytes secreting cytokines [27]. The other mechanism playing a role in the glaucoma pathogenesis are: 5—excitotoxicity [28] caused by disruptions in glutamate transport leading to apoptosis of RGCs and 6—mechanical stress connected with IOP spikes [29] that increase stiffness in the trabecular meshwork. These last two processes are unlikely to have a real effect on RGCs’ recovery in this trial, because the time of therapy was too short to let them operate.

The first reason why RGCs’ function improvement was possible is that, if restored to normal values, IOP stops deforming the tissues building ONH. Lower translaminar pressure, defined as a difference between IOP and intracranial pressure (ICP), facilitated axoplasmal flow, so the transport of intracellular nutrients, protein biosynthesis and glycolysis was possible. Such RGCs acquired a new chance to improve metabolism. We can also admit that lower IOP allowed retrograde transport of neurotrophins, which are necessary for differentiation, regeneration and development of RGCs. It is also possible that reduction in astrocytic interleukin secretion (ex. tumor necrosis factor-TNF) contributed to RGC function improvement. Cytokine biosynthesis is induced by different impulses (here increased IOP), and their half-life in the blood is short. Possibly, after IOP reduction, their concentration diminished to the level that no longer damaged RGC axons, and improvement in their function was possible.

In the presented study, the improvement in RGC function was noticed in 71% of eyes (17/24). In 29% of eyes (7/24), the PERG amplitudes did not improve. Our results show that the amplitude of the N95 wave had the most significant increase after IOP-lowering therapy, because it is represented mainly by RGCs, in contrast to the amplitude of the P50 wave, which is generated as well by cones and cone bipolar cells of the macula region [30]. Peak time of P50 did not statistically change before and after treatment. There were no interactions between the change of PERG parameters in time and type of glaucoma, as presented in Table 3. Other researchers also did not find such correlations [20]: probably, the relationship between these parameters is complex, and perhaps, the wide range of normal values for PERG test prevents creating such linear relationships. Possibly, these two glaucoma groups were too small to detect such interdependences.

In 29% of the eyes, although an IOP reduction by near 30% was achieved, the function of RGCs did not improve. The statistical differences between the group with and without improvement were not possible to establish, except lower baseline amplitudes of P50 and N95 waves in these eyes, which improved. Perhaps, the influence of IOP reduction is easier to prove in the eyes with worse function initially, because the number of dysfunctional RGCs is higher than in the eyes with PERG amplitudes around normal values. It is well known that increased IOP can influence RGC function not in the same way: an extreme example is patients with normal tension glaucoma, who suffer from glaucomatous neuropathy, despite good levels of IOP, in contrast to ocular hypertension patients, in whom the risk of developing glaucoma is only 1% per year [31].

We realize this study has limitations: the two groups are too small to draw general conclusions, and in the future, a larger sample of patients with untreated preperimetric and early-stage POAG will be recommended to test. In conclusion, our results strongly suggest that RGC function may be improved after IOP reduction by around 30%. For a chosen group of patients, hindering progression of glaucomatous neuropathy is possible through restoration of some RGCs to normal function. Wide range of therapeutic possibilities providing IOP reduction, including topical drug combinations, laser procedures and minimally invasive glaucoma surgery (MIGS), should be recommended to these selected glaucoma patients. Probably, in the group where the treatment did not improve RGC function, destruction and apoptosis of these cells is dominating, and perhaps, in these patients’ eyes, IOP-lowering therapy is less important. Pattern electroretinogram can be a useful test to monitor changes of RGC function in treated patients with POAG.
